# Application of multi-criteria decision analysis techniques and decision support framework for informing select agent designation for agricultural animal pathogens

**DOI:** 10.3389/fbioe.2023.1185743

**Published:** 2023-06-05

**Authors:** Segaran P. Pillai, Todd West, Kevin Anderson, Julia A. Fruetel, Carrie McNeil, Patricia Hernandez, Cameron Ball, Nataly Beck, Stephen A. Morse

**Affiliations:** ^1^ Office of the Commissioner, Food and Drug Administration, Silver Spring, MD, United States; ^2^ Sandia National Laboratories, U.S. Department of Energy, Livermore, CA, United States; ^3^ Science and Technology Directorate, U.S. Department of Homeland Security, Washington, DC, United States; ^4^ Sandia National Laboratories, U.S. Department of Energy, Livermore, CA, United States; ^5^ Centers for Disease Control and Prevention, Atlanta, GA, United States

**Keywords:** multi-criteria decision analysis, decision support framework, select agent designation, agriculture animal pathogen, risk assessment tool

## Abstract

The United States Department of Agriculture (USDA), Division of Agricultural Select Agents and Toxins (DASAT) established a list of biological agents and toxins (Select Agent List) that potentially threaten agricultural health and safety, the procedures governing the transfer of those agents, and training requirements for entities working with them. Every 2 years the USDA DASAT reviews the Select Agent List, using subject matter experts (SMEs) to perform an assessment and rank the agents. To assist the USDA DASAT biennial review process, we explored the applicability of multi-criteria decision analysis (MCDA) techniques and a Decision Support Framework (DSF) in a logic tree format to identify pathogens for consideration as select agents, applying the approach broadly to include non-select agents to evaluate its robustness and generality. We conducted a literature review of 41 pathogens against 21 criteria for assessing agricultural threat, economic impact, and bioterrorism risk and documented the findings to support this assessment. The most prominent data gaps were those for aerosol stability and animal infectious dose by inhalation and ingestion routes. Technical review of published data and associated scoring recommendations by pathogen-specific SMEs was found to be critical for accuracy, particularly for pathogens with very few known cases, or where proxy data (e.g., from animal models or similar organisms) were used to address data gaps. The MCDA analysis supported the intuitive sense that select agents should rank high on the relative risk scale when considering agricultural health consequences of a bioterrorism attack. However, comparing select agents with non-select agents indicated that there was not a clean break in scores to suggest thresholds for designating select agents, requiring subject matter expertise collectively to establish which analytical results were in good agreement to support the intended purpose in designating select agents. The DSF utilized a logic tree approach to identify pathogens that are of sufficiently low concern that they can be ruled out from consideration as a select agent. In contrast to the MCDA approach, the DSF rules out a pathogen if it fails to meet even one criteria threshold. Both the MCDA and DSF approaches arrived at similar conclusions, suggesting the value of employing the two analytical approaches to add robustness for decision making.

## Introduction

Incidents of biological warfare have been historically well-documented (Geissler, van Courtland Moon, 1999; Carus, 2002). While most of these incidents have been directed against humans, biological agents have also been used by state programs against animals to promote sabotage and weaken the enemy. For example, during World War I (WWI), Germany covertly inoculated military horses and cattle, most extensively those belonging to neutral suppliers of the Allied Powers, with *Burkholderia mallei* (glanders) and *Bacillus anthracis* (anthrax) (Wheelis, 1999). After WWI, many countries [e.g., Canada, France, Germany, Hungary, Italy, Japan, Soviet Union, United Kingdom (U.K.), and the United States (U.S.)] started to develop biological weapons programs primarily as a deterrent or for retaliatory purposes (Wheelis et al., 2006). Beginning in 1940, the Germans took an active interest in countering a foot-and-mouth disease (FMD) threat to their own cattle while they explored the use of this virus as an offensive weapon. Defensive vaccine production began in 1940, and by 1943 they had experimented with ways to disseminate FMD virus using little bunches of grass or hay dropped from specific heights to create an inconspicuous dispersal (Geissler, 1999). Most belligerents entered World War II (WWII) with at least exploratory biological weapons programs against personnel and animals, and most increased their activities during the war (Wheelis et al., 2006). Apparently, only the U.K. mass-produced a usable biological weapon targeting animals, which consisted of 5 million cattle cakes comprised of linseed meal laced with spores of *B. anthracis*. It was expected the cattle cakes would be dropped from bombers onto German fields to cripple their domestic animal production in retaliation if the Germans used biological weapons against the allies (Wheelis, M. et al., 2006).

After WWII, the strategic use of biological weapons against animals by state programs was, for the most part, to reduce enemy food supplies or to cause economic damage (Millett, 2006). FMD virus was the subject of considerable research as a weapon by the U.S., U.K., Canada, and the Soviet Union among others but never used (Millett, 2006; Alibek and Handelman 1999). However, there were reports describing the use of zoonotic bacterial pathogens against animal targets. In 1978, Rhodesia with assistance from South Africa purportedly attacked cattle in the Rhodesian tribal trust lands with *B. anthracis*, which also resulted in numerous human infections caused by eating infected animals or encountering spores (Mangold and Goldberg, 1999; Martinez, 2003). By 1980, more than 10,000 Zimbabweans had reportedly developed anthrax and 182 had died (Martinez, 2003). In another incident, between 1982 and 84, the Soviet Union was alleged to have attacked the mujaheddin and their horses in Afghanistan with *B. mallei* on at least one occasion (Alibek and Handelman 1999).

Today, the deliberate misuse of biological agents by terrorists and criminals poses a threat not only to public health, but also to the agricultural sector and the food chain. The intentional use of biological agents to attack crops or animal agriculture has been termed agroterrorism (Ryan and Glarum, 2008). Agriculture and food systems are extensive, open, interconnected, diverse, complex structures providing terrorists and criminals targets for plant and animal diseases. Agroterrorism is viewed as a desirable option for terrorists and criminals for several reasons. First, pathogens exist in natural reservoirs and would be relatively easy to obtain. Second, security measures at facilities where livestock are raised, or fed (i.e., feed lots) are normally low. Simple methods may be used to introduce the pathogen and the high-density conditions under which livestock are raised today, together with their mobility, will enhance its spread. Third, the time between introduction of the pathogen and when disease is noticed would allow the perpetrator to get away from the scene of the crime. Fourth, most of the animal viruses (e.g., FMD virus, Rinderpest, African Swine Fever virus) of interest to terrorists are not infectious for humans, so terrorists would not have to worry about infecting themselves. Fifth, a terrorist attack on livestock could significantly damage the U. S. economy. FMD is the most economically devastating livestock disease in the world. It has been estimated that a single case of FMD in the U. S. would result in the loss of $12–20 billion due to restrictions on cattle exports from the U. S. that would be imposed, culling of animal populations exposed to the virus, decontamination and other expenses involved in regaining national FMD-free status (Schoenbaum and Disney, 2003). Outbreaks of animal diseases, regardless of origins, could undermine the capacity to export agricultural goods, thereby generating significant losses to the economy.

Many serious animal diseases that do not exist in the U. S. (i.e., foreign animal diseases) could be of interest to terrorists and are of great concern to U. S. animal health officials. The Animal and Plant Health Inspection Service (APHIS) of the U. S. Department of Agriculture (2020) works with state animal health officials and veterinarians to identify, control, and eradicate these diseases. At the international level, the World Organization for Animal Health (WOAH, formerly the Office International des Epizooties/Epizootics [OIE]), is responsible for tracking diseases throughout the world and provides rules for animal movement and disease control. The World Trade Organization recognizes WOAH as the international agency for setting animal health standards for conducting international trade. The WOAH maintains a list of diseases of concern; the current list combines the former Lists A and B (which were mentioned in Public Law 107-188, 2002) into one consolidated list that divides the diseases of concern by host (REPORT OF THE MEETING OF THE OIE WORKING GROUP ON WILDLIFE DISEASES Paris, 4-6, 2000). Inclusion criteria for the WOAH list include four considerations: potential for international spread; significant spread within naïve populations; zoonotic potential; and emerging diseases. The presence or absence of confirmed WOAH reportable diseases in specific commercial livestock (i.e., cattle, sheep, goats, equine, swine), commercial poultry and aquaculture species are currently monitored in WOAH member states by domestic programs (e.g., National Animal Health Reporting System) (Ryan and Glarum, 2008).

So far, agroterrorism has not been a serious problem; however, the proliferation of terrorist groups with different agendas and the availability of biological agents in the environment heightens concerns (Keremidis et al., 2013). The complex global food trade and risks associated with livestock transport present vulnerabilities that may have undesirable economic animal and public (if zoonotic) health implications. Furthermore, an attack on animals is generally viewed as more restrained and less offensive than an attack against humans. Agricultural terrorism is not about killing animals; it is about crippling an economy. The outbreak of FMD in the UK in 2001 highlighted the enormous consequences, both economic and in animal health, that even a natural outbreak can have for a country (Gibbs, 2003).

These events and others have led to the promulgation of regulations to ensure the biosafety and biosecurity of animal pathogens. The effort began in 1996 when the U.S. Congress passed the Antiterrorism and Effective Death Penalty Act (Public Law 104-132, 1996) in recognition of the need for regulations to ensure the safe and secure transfer of hazardous biological agents and toxins when shipped between facilities. The legislation directed the Department of Health and Human Services (2020) to establish a list of biological agents and toxins (Select Agent Regulation. 42 C.F.R. Part 73, 2023), which included zoonotic pathogens, that could potentially threaten human health and safety. This list ultimately became part of the Select Agent Regulation, which was delegated by DHHS to be administered by the Centers for Disease Control and Prevention (CDC) (Morse, 2015). In the aftermath of the release of *B. anthracis* spores through the U.S. mail in the fall of 2001, Congress significantly strengthened and expanded oversight of Select Agents with the passage of the Public Health Security and Bioterrorism Preparedness and Response Act of 2002 (Public Law 107-188, 2002); among other things, this law expanded controls from shipment of hazardous biological toxins and agents to their possession and use. Subtitle B (Agricultural Bioterrorism Protection Act of 2002) of PL 107–188 directed the Secretary of the USDA to establish and maintain a list of biological agents and toxins that he/she determined have the potential to pose a severe threat to animal health or products. The criteria for inclusion on this list included: 1) availability and effectiveness of pharmacotherapy and prophylaxis to treat and prevent any illness; 2) economic impact; 3) inclusion on the then-OIE A and B lists (Ryan and Glarum, 2008); and 4) presence on the Australia Group List (Australia Group List, 2017). Non-biological criteria—economic consequences and effect on international trade agreements—were of paramount importance when considering agents for this list. Thus, these agents have been designated USDA Select Agents not because they necessarily pose a threat to animal health but because they pose a threat to national security (National Research Council, 2010). This contrasts with the DHHS list where the impact on public health and safety were primary factors for inclusion. Agents and toxins that appear on both the USDA and DHHS lists are referred to as Overlap Agents and are regulated by both agencies. The comparable USDA regulation 9 C.F.R. Part 121 governs select agents and toxins that have the potential to pose a severe threat to animal health or to animal products (Select Agent Regulation. 9 C.F.R. Part 121, 2023)*.* Furthermore, Title 7 U.S. Code 8401 requires the Secretary of USDA to review and republish the list biennially, or more often as needed, and shall by regulation revise the list as necessary.

Recently, we explored the applicability of MCDA techniques and DSF logic tree analyses to assist the CDC Select Agent Program’s biennial review of the Select Agent and Toxin List, applying the approach broadly to include non-select agents and toxins to evaluate its generality (Pillai et al., 2022; Pillai et al., 2022). A description of these methodologies, their advantages and disadvantages, and their prior use has been previously described (Pillai et al., 2022).

In this study we evaluated whether approaches used for HHS agents would be effective in assisting the USDA DASAT in their biennial review process. Two analytical approaches were developed and evaluated for classifying bacteria and viruses as USDA Select Agents: an MCDA framework and a DSF logic tree. Previous efforts by the USDA DASAT to review its Select Agent List relied solely on subject matter expert (SME) assessments to assess the agents and did not include non-select agent pathogens due to the additional burden placed on the SMEs. The analytical approaches we describe herein seek to provide a systematic approach and decision analysis techniques for assessing the impact on national security, and to reduce the burden on SMEs by documenting the supporting data from peer-reviewed literature in agent fact sheets to support the process.

## Methods

### Analytical framework

The starting point for the MCDA analysis was a set of 21 criteria (Table 1) that affect bioterrorism risk, including factors that would affect the public health impact of zoonoses. For convenience, these criteria were grouped into those that are relevant for agent production, agent exposure, exposure consequence, mitigation, or potential economic impact (Table 1). SMEs, or the analysis team, scored these 21 criteria on a scale of 0–10, based on the scoring definitions in Table 1, for each of the biological agents in Table 2. The scoring scale reflects relative concern as it pertains to the agent’s designation as a select agent, with 0 corresponding to lowest concern and 10 corresponding to highest concern. For simplicity, a linear scale was chosen for this evaluation. Table 1 lists the scoring definitions for each of the criteria for even-numbered scoring options (0, 2, 4, 6, 8, and 10). In the event SMEs were not in agreement on an even-numbered score, which sometimes occurred for criteria with more qualitative data, we assigned odd-numbers as an intermediate score.

**TABLE 1:** Criteria scoring definitions.

PRODUCTION	
**Ease of Production (1)**– The ease of producing agent in the laboratory as measured by the skill required, availability of growth media and equipment, time required, yield and storage stability.	
** Production Skill Required (1a)** – The level of training and agent-specific expertise needed to produce the agent and maintain pathogenicity:	
** **0	Difficult to produce
** **2	Expert-level training and agent specific experience
** **4	Expert-level training with similar organisms
** **6	Proficient in tissue culture and/or expert in aseptic technique
** **8	Basic microbiology training
** **10	Untrained
** Growth Conditions (1b)** – The availability of growth media, culture and/or equipment required to successfully grow the agent:	
** **0	No known cell lines available
** **2	Virus: Special post processing required
** **4	Virus: Special cell line required. Bacteria: Must be grown *in vivo* or *in vitro*
** **6	Virus: Common cell line required (e.g., Vero E6). Bacteria: Requires cell line or anaerobic conditions
** **8	Bacteria: Only grown in a single, complex broth or requires additional processing
** **10	Bacteria: Can be grown in common broths
** Growth Time (1c)** – The length of time to produce the agent based on growth characteristics of the agent:	
** **0	>1 month
** **2	14-28 days
** **4	10-13 days
** **6	7-9 days
** **8	3-6 days
** **10	2 days or less
** Production Yield (1d)** – Highest concentration (pfu or cfu/mL) achieved by experts using optimal production methods:	
** **0	<10^2^ per mL
** **2	10^2^-10^3^ per mL
** **4	10^4^-10^5^ per mL
** **6	10^6^-10^7^ per mL
** **8	10^8^-10^10^ per mL
** **10	>10^10^ per mL
** Storage Stability (1e)** – The amount of agent lost during storage at 4^o^C:	
** **0	>1 log loss/day
** **2	1 log loss/day
** **4	1 log loss/week
** **6	1 log loss/month
** **8	1 log loss/year
** **10	<1 log loss/year
** Ability to Genetically Manipulate or Alter (2)** – The degree of difficulty of the techniques required to create a more virulent, transmissible, environmentally stable or countermeasure-resistant strain:	
** **0	No known method to genetically manipulate and maintain pathogenicity
** **2	Very difficult (e.g., negative strand RNA viruses)
** **4	Highly difficult (e.g., positive strand RNA viruses, gene reassortment or reverse genetics available)
** **6	Moderately difficult (e.g., DNA viruses and intracellular bacteria)
** **8	Low difficulty (e.g., plasmid insertion for bacteria)
** **10	No directed genetic manipulation required (e.g., can use selection for antibiotic resistance)

EXPOSURE	
**Susceptible Hosts (3)** – Number and type of livestock species that are susceptible to the disease	
** **0	None
** **2	Horses, goats, sheep or fish
** **4	Poultry
** **6	Cattle or pigs
** **8	Multiple agricultural animal hosts
** **10	Multiple agricultural animal hosts and/or zoonotic
**Environmental Stability (4)** – The extent to which the agent is stable in the environment (outside the host) in matrices such as soil and dried on surfaces	
** **0	Agent decays immediately upon dissemination
** **2	Agent persists in indoor environments for minutes to hours
** **4	Agent persists in indoor environments for days to weeks
** **6	Agent persists in indoor environments for months to years or outdoors for hours to days
** **8	Agent persists in outdoor environments for weeks to months
** **10	Agent persists in outdoor environments for > 1 year
**Ease of Introduction (5)** – The ease with which the agent can be introduced to the target host	
** Route of Exposure (5a)** – The routes in which the disease is infectious to livestock. Routes below limited to direct contact, cutaneous, vector, ingestion, inhalation. Vertical and trans-mammary transmission not included	
** **0	None
** **2	Direct contact, cutaneous and/or vector
** **4	Ingestion
** **6	Inhalation
** **8	2 different routes
** **10	3 different routes
** Infectious dose (ID**_**50**_ **) (5b)** – The dose or amount of agent (in cfu or pfu as appropriate) required to infect 50% of a healthy livestock population by inhalation or ingestion (score worst case):	
** **0	Not infectious by inhalation or ingestion
** **2	>10,000
** **4	1000-10,000
** **6	100-1000
** **8	10-100
** **10	1-10
** Transmissibility animal to animal (5c)** – The extent to which the disease can be transmitted from one animal to another within a farm	
** **0	Non-communicable and non-transmissible
** **2	Rare animal-to-animal transmission
** **4	Transmission via non-airborne vectors such as ticks or limited animal-to-animal transmission
** **6	Moderate animal-to-animal transmission
** **8	Relatively high transmission via airborne vectors such as mosquitoes and flies
** **10	Highly transmissible among one or more animal species

CONSEQUENCES	
**Farm Production (6) --** The impact on animal/farm production (meat, eggs, milk, hides, breeding) due to illness	
** **0	Little or no symptoms or impact
** **2	Minimal to no impact on production due to mild symptoms of short duration
** **4	Decreased production for up to 1 month
** **6	Decreased production among existing herd for months to a year due to ongoing symptoms or treatments
** **8	Decreased production due to symptoms among existing herd and losses of replacement stock (e.g., abortions, neonatal mortality, sterility, inability to breed)
** **10	Total production loss due to culling and/or acute mortality with no replacement stock available
**Status of Immunity (7)** – The extent to which the population has immunity to the disease due to previous exposure or vaccination:	
** **0	Close to 100%
** **2	Majority (>80%) of population have immunity
** **4	Significant portion (20-80%) of population have immunity
** **6	Previous vaccines may have reduced impact
** **8	Small subset (<5%) have immunity
** **10	No presumed immunity to agent in population
**Acute Mortality (8)** – The number of deaths from the disease per 100 diagnosed cases (case fatality rate). Deaths are based on a non-vaccinated, sensitive population and includes deaths resultant from culling practices.	
0	Close to 0%
2	1-9%
4	10-29%
6	30-39%
8	40-49%
10	50-100%
**Transmission Farm to Farm (9)** – The extent to which the disease can be transmitted from one farm to another	
** **0	Non-communicable and non-transmissible between farms
** **2	Transmission via wildlife
** **4	Fomite transmission and/or limited farm to farm transmission observed
** **6	Vector transmission
** **8	Fomite and vector transmission
** **10	Air-borne transmission
**Public Health Impact (10)** – The potential impact on human health from the agent	
** **0	Does not cause disease in humans
** **2	Causes mild symptoms and/or is only rarely lethal in humans
** **4	Causes moderate morbidity and low mortality (CFR <9%) in humans
** **6	Causes moderate morbidity and mortality (CFR 10-29%) in humans
** **8	Causes high morbidity and mortality in humans (CFR >30%)
** **10	Causes high morbidity and mortality in humans and is human-to-human transmissible

MITIGATION	
**Availability of Vaccines (11)** – The availability of vaccines and extent to which they can be rapidly deployed and administered in response to an animal health emergency to prevent disease and transmission:	
** **0	No vaccine required (includes already vaccinated) or unlikely to be administered
** **2	Widely available and easy to deploy efficiently, e.g., a single course
** **4	Widely available but difficult to deploy efficiently, e.g., multi-course, lengthy; or lacks efficacy
** **6	Approved vaccine available in limited quantities and/or vaccine approved in other countries; available in US through IND
** **8	Experimental, unapproved vaccine in development
** **10	No vaccine available
**Farm Impact (12)** – The potential impacts to a farm due to animal quarantine, decontamination and restoration during and after an event :	
** Animal Quarantine (12a)** – The duration and extent of quarantine that may be required for animals potentially exposed to the agent:	
** **0	None
** **2	1-7 days
** **4	8-15 days
** **6	16-90 days
** **8	91-365 days
** **10	>1 year or unknown
**Decon and Restoration (12b)** – Effort required after the outbreak to return to normal operations:	
** **0	No decon required
** **2	Low level disinfectants such as quaternary ammonium compounds are effective (e.g., gram(-) bacteria, enveloped viruses)
** **4	Intermediate level disinfectants such as 70% ethanol, phenolics and iodophors are effective (e.g., gram(+) bacteria, fungi)
** **6	High level disinfectants such as glutaraldehyde, H2O2, ClO2, peracetic acid are required (e.g., non-enveloped viruses)
** **8	Extensive chemical decon and restoration is required (e.g., spores, mycobacteria)
** **10	Highly resistant to disinfection or sterilization methods

ECONOMIC IMPACT	
**Burden/ Impact on US Agriculture (13)** – The potential economic impacts to US agriculture during and after an event.	
** Export trade impact (13a)** – The value of the industry and extent to which the commodity is exported from the US as measured by percent of total US production	
** **0	No impact to US industry or food industry
** **2	US industry size is small (<$5B/yr) and not significantly exported
** **4	US industry size is small (<$5B/yr) with significant exports (>10%) or expected to be minimal due to limited animal to animal or farm to farm transmission, existing treatment, and surveillance and remedial efforts
** **6	US Industry size is large ($5-50B/yr) and not significantly exported
** **8	US industry size is large ($5-50B/yr) with significant exports (>10%)
** **10	US industry size is very large (>$50B/yr) with significant exports (>10%)
**US Industry Impact (13b)** – The scope and duration of impacts to US agriculture during and after an event:	
** **0	No impact to the Ag industry
** **2	Low impact to industry, as typically non-fatal and animals recover with little or no intervention
** **4	Short-term impact on a limited scale, due to low disease persistence and/or effective decontamination and limited farm-to-farm transmission
** **6	Longer-term impact on a limited scale, due to disease persistence and/or need for culling and limited farm-to-farm transmission
** **8	Potential for industry-wide impact due to need for culling and high farm-to-farm transmission
** **10	Significant industry-wide impact due to difficulty in eradication (e.g., high disease persistence, farm-to-farm transmission and need for culling)

**TABLE 2:** List of animal and aquaculture select, and non-select agents considered in this analysis.

Tier 1 Select Agents	Non-Select Agents
• *Bacillus anthracis* *^a^*	• Avian Influenza virus (low path) (LPAI)
• *Burkholderia mallei* *^a^*	• Bluetongue virus
• *Burkholderia pseudomallei* *^a^*	• Camel Pox virus
• Foot and Mouth Disease virus (FMD)^b^	• Getah virus
• Rinderpest Virus	• Japanese Encephalititis virus (JEV)
Select Agents	• Louping Ill virus (LIV)
• African Horse Sickness virus (AHSV)	• Malignant Catarrhal Fever virus (MCFV)
• African Swine Fever virus (ASFV)	• Menangle virus
• Avian Influenza virus (hi path) (HPAI)	• Nairobi Sheep Disease (NSDV)
• *Bacillus anthracis* Pasteur^a^	• Orf virus
• *Brucella abortus* *^a^*	• Rabies virus
• *Brucella melitensis* *^a^*	• Suid Herpesvirus 1 (SHV1)
• *Brucella suis* *^a^*	• Vesicular Stomatitis virus (VSV)
• Classical Swine Fever virus (CSFV)	• Infectious Hematopoietic Necrosis virus^c^ (IHNV)
• Hendra virus^a^	• Infectious Salmon Anemia virus^c^ (ISAV)
• Lumpy Skin Disease virus (LSDV)	• Spring Viremia of Carp virus^c^ (SVCV)
• *Mycoplasma capricolum*	• Viral Hemorrhagic Septicemia virus^c^ (VHSV)
• *Mycoplasma mycoides*	
• Newcastle virus	
• Nipah virus^a^	
• Peste des Petite Ruminants virus (PPR)	
• Rift Valley Fever virus^a^ (RVFV)	
• Sheep and Goatpox virus (S&G Pox)	
• Swine Vesicular Disease virus (SVDV)	
• Venezuelan Equine Encephalitis virus^a^ (VEEV)	

^a^ Overlap Select Agents.

^b^ Abbreviations used in Figures.

^c^ Aquaculture pathogens.

**FIGURE 1:**
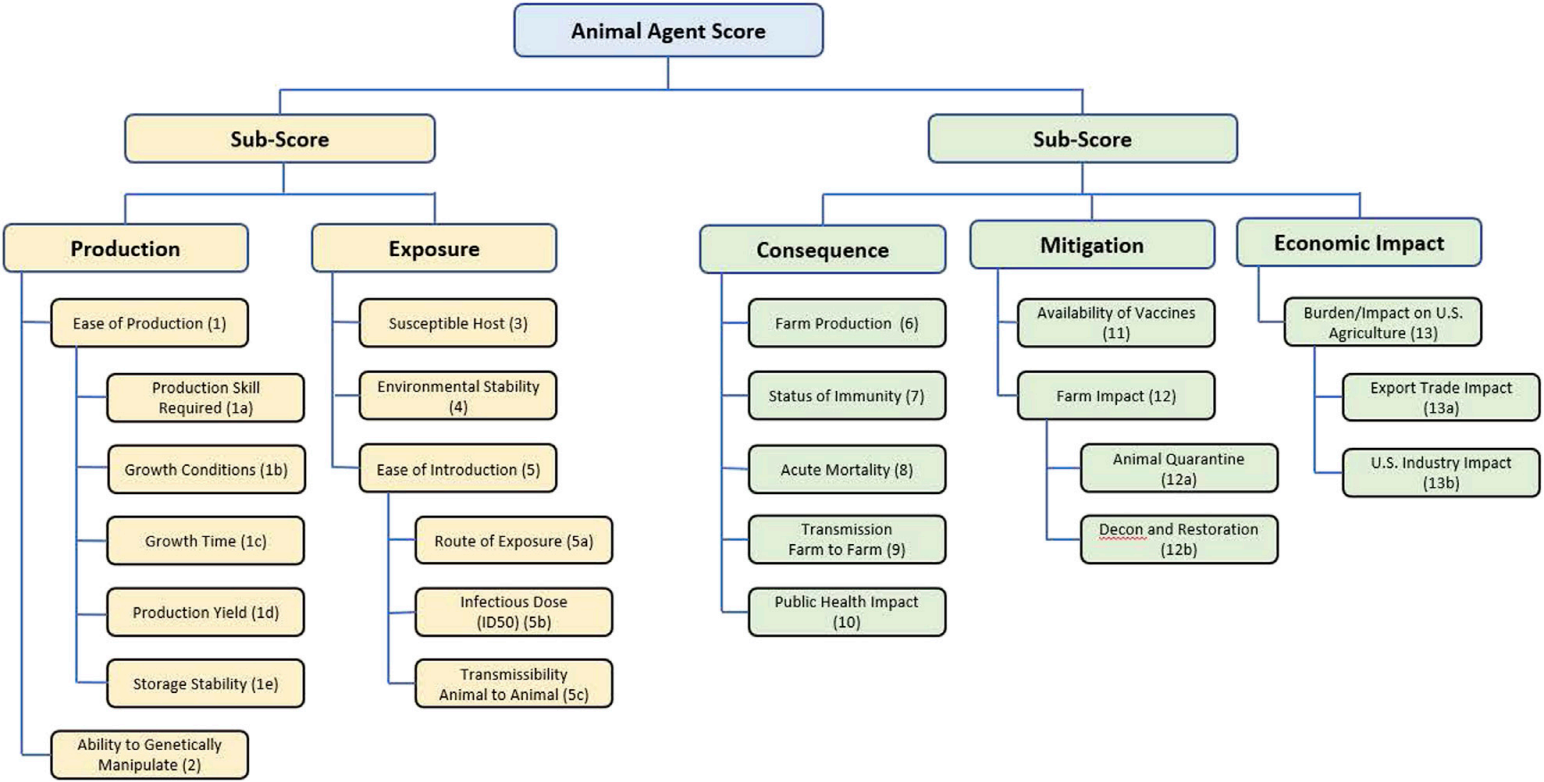
Summary of the criteria and hierarchy captured in the MCDA tool and fact sheets for animal select agent tiering.

The scores for each agent were used to inform identification of pathogens for consideration as select agents as follows. Several of these scores had multiple components: first, scores for 1a, 1b, 1c, 1d and 1e (Table 1) were averaged to give a score for Ease of Production (Criterion 1); scores for 5a, 5b and 5c were averaged to give a score for Ease of Introduction (Criterion 5); scores for 12a and 12b were averaged to give a score for Farm Impact (Criterion 12); and scores for 13a and 13b were averaged to give a score for Burden/Impact on US Agriculture (Criterion 13) as succinctly summarized in Figure 1.

Next, the resulting 13 factor scores, i.e., the four composite scores noted above (1, 5, 12, and 13) plus the remaining nine single-criterion scores (2, 3, 4, 6, 7, 8, 9, 10 and 11) for each biological agent were compiled in two ways: 1) a one-dimensional (1-D) ranking whereby the total unweighted or weighted sum (as defined in the next section) for each agent was tallied and the agents were ranked from lowest to highest; and 2) a two-dimensional (2-D) plot whereby the unweighted or weighted sum of the sub-scores for the “production” (1 + 2) plus “exposure” (3 + 4 + 5) branches of the hierarchy were plotted against the unweighted or weighted sum of the sub-scores for the “consequences” (6 + 7 + 8 + 9 + 10) plus “mitigation” (11 + 12 + 13) branches of the hierarchy (see Figures 4, 6).

### Criteria weighting

Weights were assigned to each criterion to account for factors that may carry more significance for the goals of the select agent program. SMEs ranked each of the 13 criteria collectively, from one to three, where one described the least important criteria and three described the most important criteria. To demonstrate the MCDA methodology, two weighting schemes were tested: equal weighting, i.e., unweighted and the weighting scheme derived from the SME’s inputs, as shown in Table 3. In the latter case, seven criteria (Ease of Production, Ease of Introduction, Farm Production, Status of Immunity, Acute Mortality, Transmission farm-to-farm and Burden/Impact on U.S. Agriculture) were given a 3x weight, two criteria (Availability of Vaccines and Farm Impact) a 2x weight, and the last four criteria (Ability to Genetically Manipulate, Susceptible Host, Environmental Stability and Public Health Impact) a 1x weight. For both cases, criteria and weights were combined into a single score A) by summing all the weighted numerical values (a_ij_,w_i_), where a_ij_ represents a criteria score and w_j_ is the criteria weighting value:

$$A=\sum_{j=1}^{n}aij\;•\;wj$$

**TABLE 3:** Proposed weighting schemes explored for animal select agent tiering.

Criteria	**SME assigned weight**
1) Ease of production	3
2) Ability to genetically manipulate	1
3) Susceptible hosts	1
4) Environmental stability	1
5) Ease of introduction	3
6) Farm production	3
7) Status of immunity	3
8) Acute mortality	3
9) Transmission farm-to-farm	3
10) Public health impact	1
11) Availability of vaccines	2
12) Farm impact	2
13) Burden/Impact on US agriculture	3

To enable comparison of results using different weighting values, normalized scores were used, whereby the total or sub-total scores were normalized to those of a hypothetical agent that received 10s for all 21 criteria scores.

### Agent fact sheets

To document the data used for scoring pathogens against the 21 criteria noted above, we developed agent fact sheets for 41 pathogens (Table 2). The list includes 24 USDA select agents, of which 11 are also HHS Select Agents (i.e., overlap agents), and 17 non-select agents, of which 4 aquaculture pathogens were included in the analysis based on SME input.

Development of the agent fact sheets used peer-reviewed open literature such as Medline, PubMed, Google Scholar and other unclassified data followed by extensive review by SMEs who work with the specific pathogen. In situations where there were data gaps, SME judgment provided a basis for scoring, referencing data for similar organisms or relevant models as appropriate (e.g., laboratory challenge experiments for infectious dose). In circumstances where a range of values was found (e.g., production yields, infectious dose), the worst reasonable case (i.e., leading to the largest “bad” outcome) was typically used for scoring. In all cases, SME judgement was relied upon to provide concurrence on the best available data or basis for scoring. SMEs identified by the USDA DASAT were asked to review the data provided on the fact sheets for accuracy and relevance, as well as the scores assigned to each data category. Comments received from SMEs were verified through literature search, review of unpublished data and corroboration with other SMEs and incorporated into the agent fact sheets and scoring adjusted, as necessary.

### Decision support framework (DSF)

**FIGURE 2:**
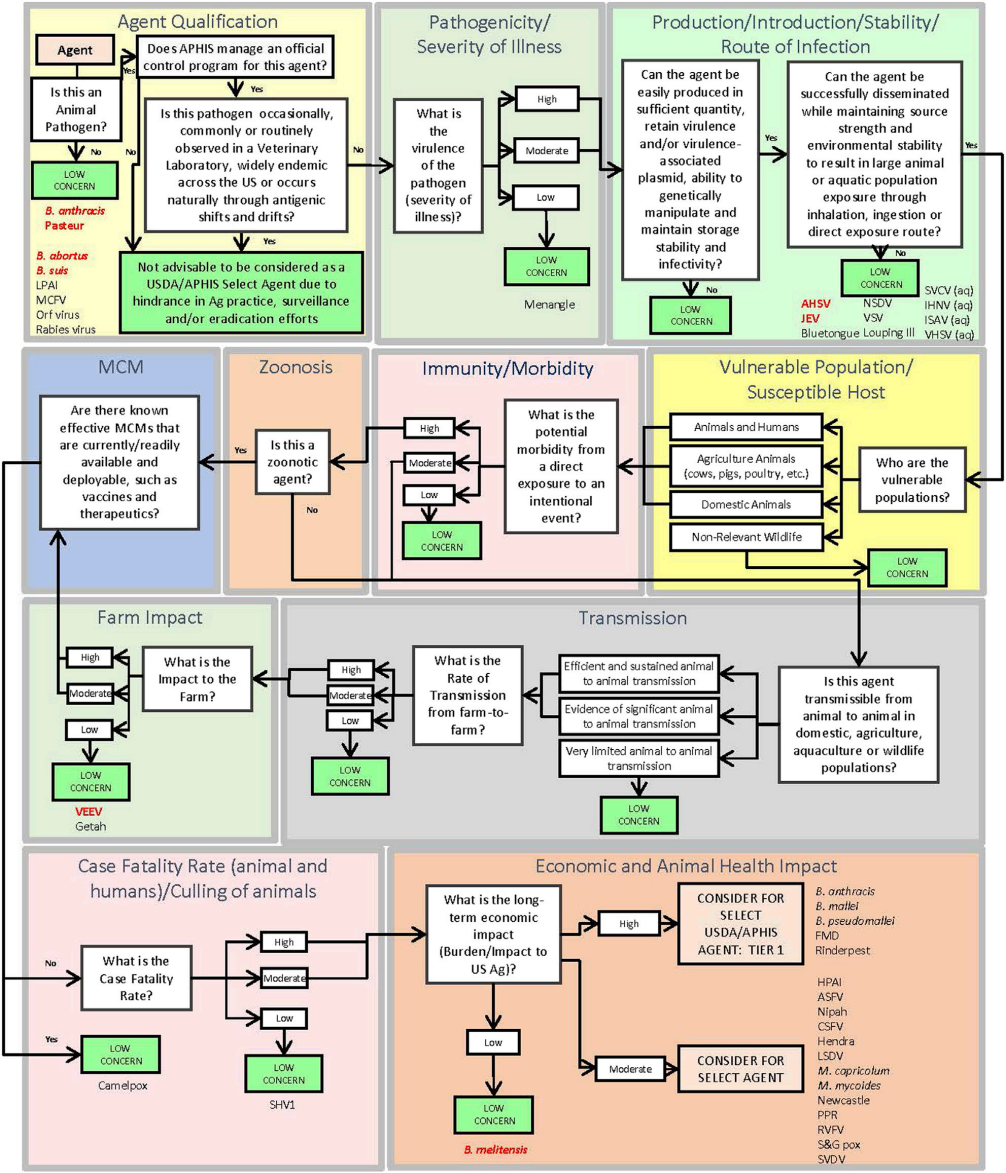
Schematic of the Decision Support Framework logic tree showing assignment of animal select and non-select agents (Abbreviations as in Table 2).

The DSF approach applies key criteria using a logic tree format to identify pathogens which may be of sufficiently low concern that they can be ruled out from consideration as a select agent. The DSF is complementary to the MCDA approach and avoids the possible unintended numerical equivalences that may occur using weighted, or unweighted, sums. Additionally, the DSF considers the potential impact associated with regulating an agent versus the agricultural implications and animal health practices. Using the DSF approach as shown in Figure 2, if a pathogen does not meet a threshold value for any one of the criteria set, it is deemed of low concern and thus is not considered for select agent status. Those pathogens that exceed all criteria thresholds are considered for select agent status. Criteria include Agent Qualification, Pathogenicity/Severity of Illness, Production/Introduction/Stability/Route of Infection, Vulnerable Population/Susceptible Host, Immunity/Morbidity, Zoonosis, Transmission, Farm Impact, Medical Countermeasures, Case Fatality Rate (animals and humans if zoonotic)/Culling of animals, and Economic and Animal Health Impact. SME judgment based on data captured in the agent fact sheets provided the basis for scoring. In general, criteria which received a score of zero, two or four in some cases typically served as a basis for a “low concern” qualitative assessment. In contrast to the MCDA approach, which uses a graded scoring system for ranking agents, the DSF approach can rule out an agent from select agent consideration using a single (low scoring) criterion. Many of the criteria overlap between the MCDA and DSF approaches.

## Results

### Data gaps and quality

When considering many micro-organisms across a broad range of attributes, data gaps and variability in data quality are inevitable. Data availability in the open literature tended to parallel scientific inquiry for the organism; for example, aerosol studies were more prevalent for pathogens known or suspected to be infectious by the aerosol route, and surface stability data were generally more available for pathogens where fomite transmission is a concern. Overall, we found the most prominent data gaps were in aerosol stability and animal infectious dose by inhalation and ingestion routes. For aerosol stability data, we typically used data for similar organisms (e.g., same virus family) as proxies, and infectious dose data from animal models where available to address data gaps.

### Unweighted rankings

**FIGURE 3:**
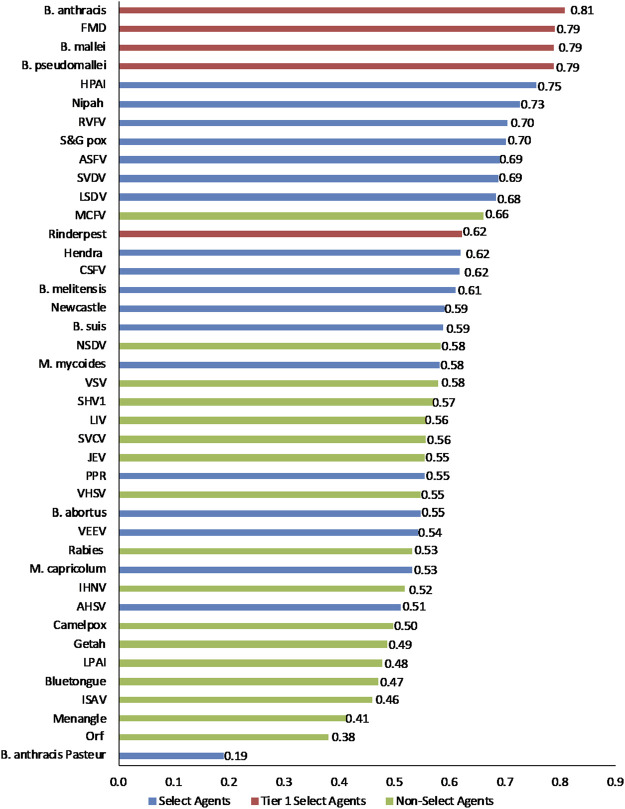
1-D plot of unweighted scoring results for animal select agent tiering (Abbreviations as in Table 2).

**FIGURE 4:**
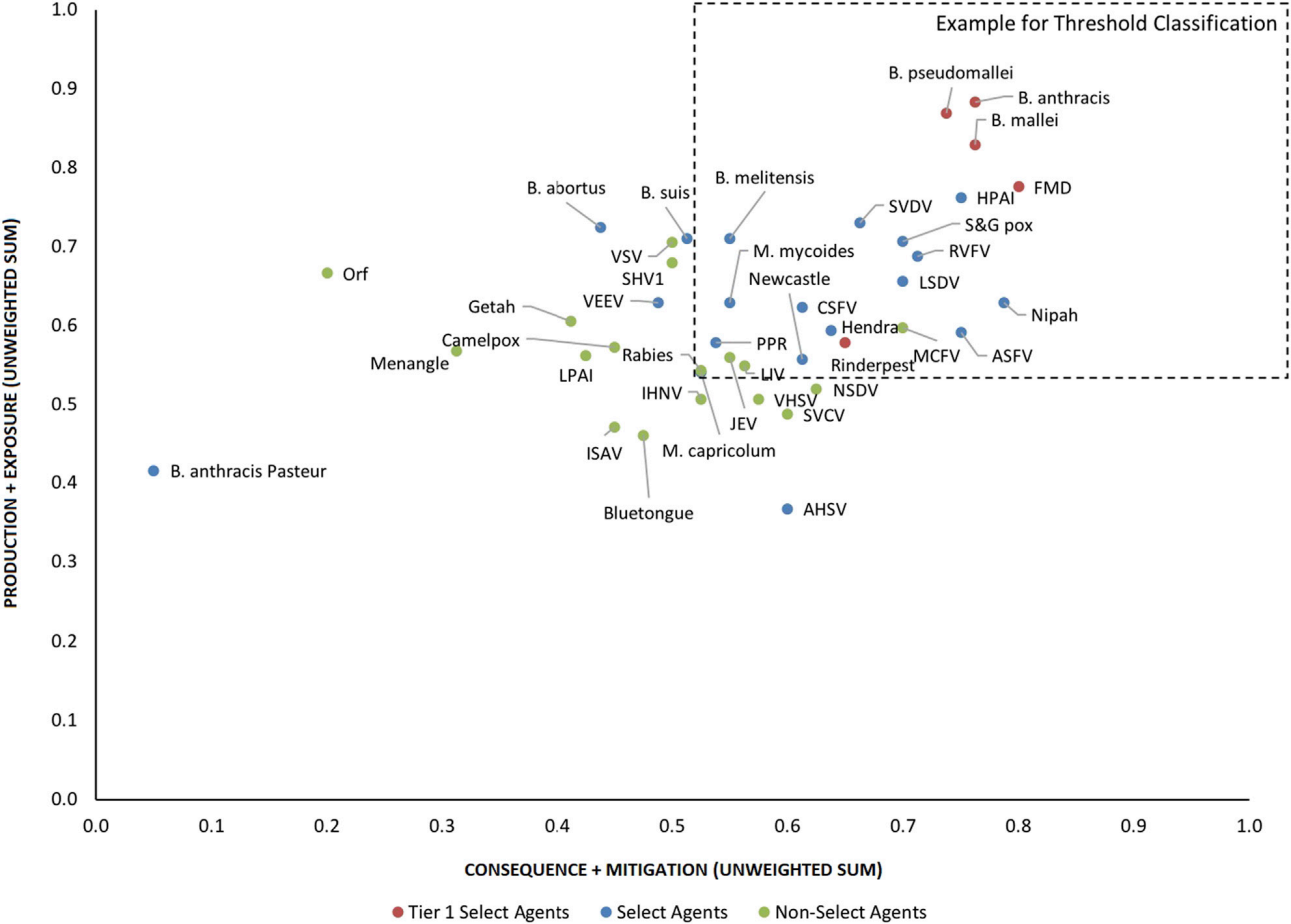
2-D plot of unweighted scoring results for animal select agent tiering (Abbreviations as in Table 2).

**FIGURE 5:**
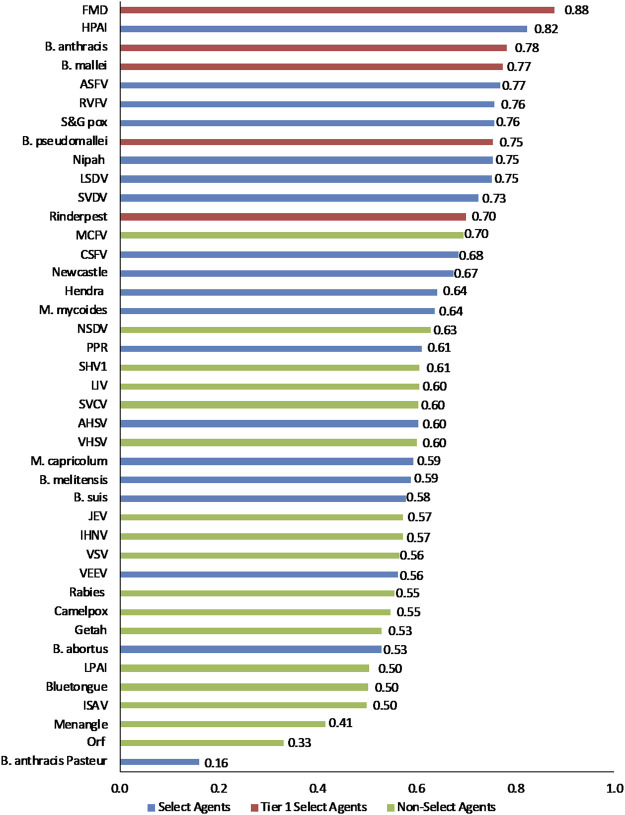
1-D results for the proposed weighting scheme for animal select agent tiering (Abbreviations as in Table 2).

**FIGURE 6:**
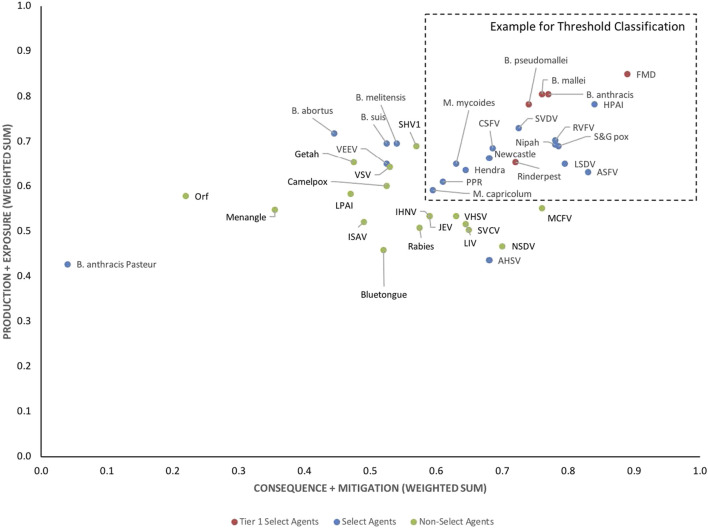
2-D results for the proposed weighting scheme for animal select agent tiering (Abbreviations as in Table 2).

To facilitate comparison of the analytical results with current assignments as Tier one select agents, select agents, and non-select agents, the three classes of agents were color coded red, blue and green, respectively, in Figures 3–6.

Initial inspection of the 1-D results, whereby the total summated scores for all 41 pathogens are compared (Figure 3) indicated that, in general, the Tier 1 select agents were found at the top of the rank-ordered list, other select agents fell in the middle section, and non-select agents comprised the bottom section; however, there were exceptions. Similarly, for the 2-D plots, whereby summated sub-scores for all 41 pathogens are plotted against each other (Figure 4), Tier 1 select agents and other select agents were generally found in the upper right quadrant of the plot, while non-select agents generally fell outside that area; however, there were exceptions.

Analysis of both the 1-D and 2-D plots indicated that, although there were general trends in the data that were consistent with current classifications, there were no sharp breaks or gaps in scoring that would serve as a basis or threshold for classifying an agent as a select agent. Instead, the plots represented a continuum of scores. Additionally, any designation of a minimal score—whether the total score in the 1-D plot, or sub-scores corresponding to the x- and y-values in the 2-D plots—resulted in some exceptions to current classifications. While the current Select Agent List is not absolute nor the definitive source for which agents should be considered select agents, it provides a useful reference point for evaluating the impact of setting minimum scoring thresholds as the basis for classifying pathogens as select agents.

For example, in the 2-D plot, if the threshold for the x-axis and y-axis scores for a select agent were designated as 0.53 and 0.54, respectively, based on SME input, this led to the notional threshold for classification as shown in Figure 4. Using this basis for classification, we found that all current select agents reclassified as select agents except African Horse Sickness virus, *B. anthracis* Pasteur, *B. abortus*, *B. suis*, and Venezuelan Equine Encephalitis virus. All non-select agents reclassified as non-select agents except Japanese Encephalitis virus, Louping Ill virus, Malignant Catarrhal Fever virus*,* and Rabies virus.

### Weighted rankings

The data using the proposed weighting scheme in Table 3 for 1-D and 2-D formats are shown in Figures 5, 6, respectively. As observed with the unweighted data, the general trend in the data was consistent with current classifications; however, any designation of a minimal score as a basis for classification—whether the total score in the 1-D plot, or sub-scores corresponding to x- and y-axes values in the 2-D plots—resulted in some exceptions to current classifications. For example, in the 2-D plot, if we designated the lowest *x*-axis and y-axis scores allowed for classification as a select agent to be 0.59 and 0.58, respectively, based on SME input, as illustrated in Figure 6, we found that all select agents reclassified as select agents except African Horse Sickness virus, *B. anthracis* Pasteur, *B. abortus*, *B. melitensis*, *B. suis*, and Venezuelan Equine Encephalitis virus. All non-select agents reclassified as non-select agents.

### Decision support framework

To evaluate the 41 select and non-select agents using the DSF approach, we leveraged the agent fact sheets developed for this analysis. For the factor of Pathogenicity/Severity of Illness, the score for Farm Production was used as it incorporates the clinical information affecting diseased animals, with a score of 2 or below used to determine low level of concern. A score of 4 or below for Ease of Production was used to determine low level of concern for production. Ease of Introduction was used to determine Introduction, Stability, and Route of Infection with a score of 4 or below to determine agents of low concern. A score of 0 for Vulnerable Population and Susceptible Host was used to determine an agent was of low concern. A score of 0 for Immunity and Morbidity was used to determine an agent was of low concern. A score of 0 for animal-to-animal transmission was used to determine the low level of concern. A score of 0 for Transmission Farm-to-Farm was used to determine an agent was of low concern. A score of 2 or below was used for Farm Impact to determine an agent was of low concern. A score of 0 for availability and effectiveness of medical countermeasures was used to determine an agent was of low concern. A score of 4 or below for Case Fatality Rate and Culling of Animals by leveraging Acute Mortality data to determine an agent was of low concern. A score of 2 or below for Economic, and Animal Health Impact was used to determine an agent was of low concern. The results (Figure 2) showed that all select agents were identified for consideration as select agents except African Horse Sickness virus, *B. anthracis* Pasteur, *B. abortus*, *B. melitensis*, *B. suis*, and Venezuelan Equine Encephalitis virus. All non-select agents were ruled out from select agent consideration.

## Discussion

Pathogen selection and prioritization for a specific intended use could be carried out using a formalized risk ranking process with weighted criteria that were selected to meet a required objective (McFadden et al., 2016). Similar processes have been previously used in both public health and veterinary health spheres (Caroden et al., 2009; Havelaar et al., 2010; Ciliberti et al., 2015; McFadden et al., 2016; Roelandt et al., 2017) to support prevention, early warning surveillance and control measures for disease incursion. Although there is no universal methodology for risk ranking, it is important that risk ranking exercises use a structured approach, which is transparent and consistently documented to be reproducible. MCDA- and DSF-based risk assessments are already recognized as useful tools to support select agent and toxin designations (Pillai et al., 2022; Pillai et al., 2022).

Here we investigated using MCDA and DSF as a structured approach to inform the designation of select agents of agricultural significance. The approach was flexible with the ability to adjust both the criteria and their weighting based on SME input and contribution.

The criteria we employed in this analysis are based on those identified in the Agricultural Bioterrorism Protection Act of 2002 Part B (Public Law 107-188, 2002), which directs the USDA Secretary to establish and maintain a list of biological agents and toxins that he/she determined have the potential to pose a severe threat to animal health or products. In addition, Title 7 U.S. Code 8401 requires the evaluation of whether such inclusion would have a substantial negative impact on the research and development of solutions for the animal and plant disease caused by the agent or toxin; and whether the negative impact would substantially outweigh the risk posed by the agent or toxin to animal or plant health if it is not included on the list. Comparison of these criteria with other published methods shows that many of them overlap, such as morbidity and mortality, route of exposure, environmental stability, transmissibility, ease of production, availability of Medical Countermeasure (MCMs), etc. We also include the Public Health Impact based on SME input to capture potential zoonotic impacts. Note that while it is considered an additional risk factor, zoonotic potential in and of itself would not be enough to push an otherwise low-scoring animal pathogen above thresholds for consideration as an agricultural select agent. Criteria we did not consider include public perception or terror factor, accessibility of agent and ease of detection, surveillance and laboratory diagnosis.

In addition to the choice of criteria, the focus on agroterrorism (i.e., aerosol or food-based introduction through animal feed) attacks affecting a large segment of the agricultural animal population is embodied in the scoring scales. Common pathogens causing mild illness and where there are treatments readily available may be unlikely to require a large-scale agricultural health response.

We evaluated two methods, MCDA and DSF, for their individual merits and to provide confirmation of the observed results. While both methods enabled a risk-informed comparison of a diverse set of pathogens in a structured way, the MCDA results were challenged by a continuum of scores that did not suggest natural thresholds for classification of select agents. Potential pitfalls of MCDA techniques are described in Cox et al., 2005, and while alternative treatments of the data may be of future interest (see for example, Pillai et al., 2022), this analysis highlights some of the challenges that can arise when considering a large, diverse set of pathogens. Alternatively, the DSF employs a series of criteria thresholds to identify pathogens for consideration as a select agent and provides clear classification assignments.

The finding that both approaches arrived at a consistent set of pathogens for consideration as select agents supported their usefulness. Interestingly both approaches also arrived at a consistent set of current select agents that should not be considered as select agents. The MCDA and DSF methodologies supported all current DASAT animal select agent designations and all non-select agents (Table 2) except for *B. anthracis* Pasteur*, B. abortus, B. melitensis, B. suis,* African horse sickness virus and Venezuelan equine encephalitis virus, which are currently select agents but failed to meet the criteria established for MCDA and DSF methods.

With African Horse Sickness Virus, the DSF factors related to the production and dissemination of the virus resulted in USDA SMEs concurrence that difficulties exist in the successful dissemination and effective transmission of the virus that will result in a large animal population exposure. MCDA factors that contributed to the outcome were the existence of an efficacious vaccine along with low to moderate environmental stability and difficulties associated with the introduction to an animal population and to maintain sustained transmission.

With *B. anthracis* Pasteur, the primary DSF criteria that it is not an animal pathogen indicated this agent does not qualify as a USDA select agent. MCDA factors similarly showed the agent was of no risk to farm production, mortality, farm to farm transmission, economic impact, and low risk to farm impact. In addition, the low virulence of the agent provided additional supporting data for supporting removal of the agent from the Select Agent List.

During the analysis of *B. abortus* using the DSF, it was recognized that the agent is occasionally observed in veterinary diagnostic laboratories in endemic areas, is widely distributed in wildlife hosts such as the Bison and Elk populations in Yellowstone National Park and continues to increase in prevalence and distribution. As such, inclusion of the agent on the Select Agent and Toxin list would have a substantial negative impact on the research and development of solutions for the animal disease. MCDA factors that contributed to the outcome were the existence of an efficacious vaccine, moderate immunity status of vulnerable population, limited Farm-to Farm transmission risk and moderate farm impact, and moderate risk due to difficulty related to large-scale introduction to an animal population. Economic impact was considered to have low risk from a domestic and international trade perspective due to limited Farm-to-Farm transmission, and factors that would be more regional or local to an infected premises. Public health impact was considered as a low risk with efficient treatment methods available and very low untreated mortality rates which can range from 0.5%–5% with an average of <2% (WHO guidance, 2004) and treated mortality rate is <1% (Castano et al., 2017); and in the U.S. is close to 0% (CDC, personal communication).

*B. suis* was ruled out for consideration as a select agent using the DSF because the agent is occasionally observed in veterinary diagnostic laboratories and is widely endemic in animal populations, such as feral swine population in more that 40 U.S. states, and continues to pose a significant threat to domestic swine population across the U.S. As such, inclusion of the agent on the Select Agents and Toxins list would have a substantial negative impact on the research and development of solutions for the animal disease. MCDA factors that supported removal as a select agent were the limited Farm-to-Farm transmission risk and moderate farm impact, and moderate risk due to difficulty of large-scale introduction to an animal population. Economic impact was considered to have low domestic and intentional trade risk due to limited Farm-to-Farm transmission, and factors would be more regional or local to an infected premises. Public health impact was considered as a low risk with efficient treatment availability and very low untreated mortality rate which can range from 0.5%–5% with an average of <2% (WHO guidance, 2004) and treated mortality rate is <1% (Castano et al., 2017); and in the U.S. is close to 0% (CDC personal communication).

During the analysis of *B. melitensis* using the DSF, the agent was ruled out for consideration as a select agent due to low concern associated with long-term economic and animal health impact. The effect upon agricultural economic factors was low based on the size of the domestic goat and sheep industry. MCDA factors that supported removal as a select agent were the limited Farm-to-Farm transmission risk and moderate farm impact, and moderate risk due to difficulty of large-scale introduction to an animal population. Economic impact was considered to be a low risk from the perspective of domestic and international trade, and factors would be more regional or local to an infected premises. Human infections could readily be treated with antibiotics administration with a case fatality rate close to 0% (CDC, personal communication) in the U.S.

In the case of Venezuelan Equine Encephalitis, during the DSF analysis, the agent made it through the decision tree until Farm Impact where it was recognized that an efficacious vaccine existed for this agent. Based upon the vaccine contributing to a high population immunity, the agent was considered a low concern within this category. MCDA factors that supported removal as a select agent were difficulties in large-scale production and efficient dissemination due to low environmental stability of the agent. Farm-to-Farm transmission risk was considered moderate with Farm Impact considered a low risk due to the availability of an efficacious vaccine.

Both the DSF and MCDA provide support for the recommendation to remove these agents from the USDA Select Agent List and are consistent with the 2020 proposal by the DASAT to delist *B. abortus*, *B. melitensis*, *B. suis*, *B. anthracis* Pasteur strain, Venezuelan equine encephalitis virus and African horse sickness virus (APHIS, USDA, 2020 (Federal Register Vol. 85, No. 52, 2020).

Interestingly, when equal weighting was applied across the board for all criteria, *B. melitensis* scored above the threshold for a select agent, as did JEV, Louping Ill virus, Malignant Catarrhal Fever virus, and Rabies virus. However, those agents were below thresholds using the SME-proposed weighting scheme and thresholds, and were ruled out using the DSF approach, suggesting the value of employing the two analytical approaches to add robustness for decision making.

Application of the methodology across a large and diverse pathogen set, while helping to demonstrate the robustness of the approach, highlighted the challenge of how to handle data gaps for many pathogens. At times, the use of proxies and other assumptions artificially elevated some pathogens, requiring SME review of the data and discussions on how to account for the uncertainties in the data. Thus, we found that the methodology was also useful for identifying those parameters and pathogens where more data are needed, to help with prioritizing future research studies.

## Conclusion

The goal of this effort was to explore the use of MCDA and DSF logic tree approaches for supporting the USDA DASAT biennial review. We found the use of two methods with different approaches for identifying pathogens for consideration as select agents provided robustness and benefit to support their intended use and application. The two-dimensional MCDA approach provided a risk-informed assessment that implemented the DASAT’s decision criteria and its focus on bioterrorism scenarios with the potential for large-scale agricultural health and economic consequences. The DSF is a complementary approach to identifying select agents and provided additional insight into the factors that influence decision making. The two methods represent different ends of a spectrum for using criteria thresholding to identify select agents: the MCDA approach applies thresholds after considering 21 criteria, while the DSF approach applies thresholds at the single criterion level for nine criteria. Applying weights using the MCDA approach can be used to fine-tune the effective number of criteria used to identify a threshold.

Comparison of the analytical results with the current Select Agent List provided a useful reference point for evaluating these approaches and their potential impact on decision making. Weighted data performed better at reclassifying agents with current designations than did the unweighted data. The 2-D approach most closely replicated current designations. However, the closeness of some agents to the notional threshold suggested that the results were sensitive to where the threshold line was drawn and may be sensitive to how the weights were chosen.

Overall, almost 75% of the agents evaluated classified consistently with their current designations (either select agent or non-select agent), regardless of the method chosen. Both approaches reclassified African Horse Sickness virus, *B. anthracis* Pasteur, *B. abortus*, *B. suis*, and Venezuelan Equine Encephalitis virus as non-select agents. Furthermore, the regulation described in Title 7 U.S. Code 8401, requires that the cost of continued listing and the impact to scientific advancement in research and solutions be considered. *Brucella* species create a financial burden on the federal government, States and livestock producers as we continue to mitigate the disease risk to livestock. Montana spends over 7.5 million dollars of State and Federal funds each year on *Brucella* risk mitigation (A Report to the Montana Legislature, 2017). Cost associated with the effective eradication of swine and bovine brucellosis in the U. S. between 1934 and 1998 are conservatively estimated to be over $3.5 billion (Roberts et al., 2012). Removal of *Brucella* species, from the Select Agents and Toxins list will allow for more scientists and entities to engage in the necessary research to develop tools (better vaccines, therapeutics, diagnostics, surveillance tools, containment measures etc.) needed to stop the spread and contain the disease. It is conceivable that without these tools, *B. abortus* could 1 day be found in wild elk and bison in every habitat in nearly every Western State, which is a risk to the domestic cattle population across the U.S. Similarly, *B. suis* could eventually spread through every state in the U.S. and spill over into the domestic swine population. The public health impact of *B. suis* was considered low risk because Human-to-Human transmission is very rare (Brucellosis- World Health Organization, 2020), infected wildlife in the U. S. often come in contact with humans without significant transmission (WHO guidance, 2004, and Mantur et al., 1996), effective treatment is available (such as combinations of rifampicin, streptomycin, trimethoprim-sulfamethoxazole, doxycycline, tetracycline, gentamycin, ofloxacin or ciprofloxacin) (Pappas et al., 2006), it has a long incubation period (ranging from 5 days to 6 months with an average onset of 2–4 weeks) (CDC- Brucellosis Reference Guide, 2017) and also has a long window of opportunity to treat brucellosis for a positive outcome after presentation of clinical symptoms (unlike anthrax and plague), and has a very low untreated mortality rate, which can range from 0.5%–5% with an average of <2% (WHO guidance, 2004) and treated mortality rate with <1% (Castano et al., 2017) and in the U.S. is close to 0% (CDC, personal communication). Also, *B. suis* was weaponized by the U. S. in the 1950s as an incapacitating agent and not as a lethal agent (Pappas et al., 2006). As such, removing *Brucella* species from the select agents and toxins list would pose no more risk to the Nation than that currently existing with *Brucella* species being endemic in many animal populations and being widely distributed across the U.S. with the potential for spill over to domestic cattle and swine population and secondary risk to farmers.

Throughout this process the members of Agricultural Intragovernmental Select Agents and Toxins Technical Advisory Committee and the SMEs they identified were key to providing input on the methodology and associated agent fact sheets. There are still some data gaps in the agent fact sheets, such as relevant quarantine data for some agents, that represent opportunities for further research. Regardless of these gaps, it should be noted that these agent fact sheets are meant to evolve as new data become available, from research or additional outbreaks. The MCDA and DSF represent a data driven approach for pathogen prioritization. However, it should also be noted that this methodology should not be used in a vacuum but as one component of a larger regulatory and policy decision framework.
